# Assessing the impact of the COVID-19 pandemic on recognition and testimony memory: differential effects in young and older adults

**DOI:** 10.3389/fpsyg.2025.1557634

**Published:** 2025-10-28

**Authors:** Matias Bonilla, Pablo E. Flores Kanter, Vanessa Vidal, Zahira A. Jiménez, Candela S. Leon, Facundo A. Urreta Benitez, Luis I. Brusco, Aylin Vázquez Chenlo, Yohann Corfdir, Cristian García Bauza, Cecilia Forcato

**Affiliations:** 1 Laboratorio de Sueño y Memoria, Departamento de Ciencias de la Vida, Instituto Tecnológico de Buenos Aires, Buenos Aires, Argentina; 2 Consejo Nacional de Investigaciones Científicas y Tecnológicas (CONICET), Buenos Aires, Argentina; 3 Department of Psychology, Universidad Siglo 21, Córdoba, Argentina; 4 Innocence Project Argentina, Buenos Aires, Argentina; 5 Centro de Neuropsiquiatría y Neurología de la Conducta (CENECON), Buenos Aires, Argentina; 6 PLADEMA, Universidad Nacional del Centro, Buenos Aires, Argentina

**Keywords:** episodic memory, aging, free recall, anxiety, depression, sleep, COVID-19 pandemic

## Abstract

The COVID-19 pandemic negatively impacted global mental health, with younger adults showing higher levels of anxiety and depression than older adults. Given the strong association between emotional states, sleep quality, and memory, the pandemic provided a unique context to investigate how stress influences episodic memory across age groups. We hypothesized that the typical memory advantage of younger adults would be diminished, or even reversed, relative to the performance of older adults on different memory tasks. A total of 159 participants from Buenos Aires were recruited and divided into independent samples. Younger adults during the pandemic (*n* = 42, *M* = 16.93, SD = 1.85) and post-pandemic (*n* = 38, *M* = 17.31, SD = 1.74), and older adults during the pandemic (*n* = 41, *M* = 71.36, SD = 4.84) and post-pandemic (*n* = 38, *M* = 65.38, SD = 4.03). In two online sessions, participants completed questionnaires on anxiety, depression, and sleep, watched an aversive video, and performed free recall, facial recognition, and chronological order tasks. Free recall reports were further examined with semantic network measures. Results showed that younger adults reported higher anxiety and depression than older adults, with anxiety decreasing only post-pandemic (*p* < 0.001). During the pandemic, older adults recalled more episodic details than younger adults (*p* < 0.01); however, contrary to our expectations, post-pandemic the typical pattern was not restored, as younger adults performed at the same level as older adults on this task. Younger adults performed better than older adults in recalling gist details, defined as a predefined set of central elements from the event, post-pandemic (*p* < 0.01), and consistently showed better facial recognition across both periods (*p* < 0.05). Semantic networks were more modular in older adults (*p* < 0.001), while younger adults’ networks became more efficient post-pandemic. These findings suggest that pandemic stress temporarily reversed age-related memory patterns.

## 1 Introduction

Episodic memory refers to the ability to remember personally experienced events situated in a specific time and place (Berntsen and Rubin, 2012). The formation of such memories is a dynamic process that involves several stages. New information, for example, an event, is first encoded, after which the memory trace remains in a labile state before undergoing stabilization and reorganization (consolidation). Once consolidated, the information can be retrieved and gradually integrated into cortico-cortical networks (Dudai et al., 2015; Feld and Diekelmann, 2015).

Episodic memory formation is altered because of natural aging, mainly due to declines in hippocampal and prefrontal integrity, reduced connectivity and neurochemical support, which lead to difficulties in associative binding, strategic control, and increased reliance on gist-based retrieval (Shing et al., 2010; Nyberg et al., 2012). Although some studies emphasize retrieval difficulties in aging, particularly in tasks requiring executive control (Cadar et al., 2018; Kirk and Berntsen, 2018), several lines of evidence indicate that encoding processes are especially vulnerable. Electrophysiological and behavioral studies show that age-related memory decline is better explained by deficits during encoding than by retrieval (Glisky et al., 2001; Friedman et al., 2007; Dennis et al., 2008; Suzin et al., 2019). Consistent with this literature, our own findings (Tassone et al., 2020) suggest that encoding is the most affected stage in older adults. Nonetheless, deficits have also been observed in consolidation (Kukolja et al., 2016; Muehlroth et al., 2020) and in retrieval under highly demanding tasks (Wagnon et al., 2019; Korkki et al., 2020). Furthermore, studies have shown that semantic networks in older adults tend to be more modular, comprising sets of words or nodes that are more scattered and less interconnected, and also more segregated, with greater separation between word pairs compared to younger adults (Dubossarsky et al., 2017; Siew et al., 2019b; Cosgrove et al., 2021; Wulff et al., 2022). These differences in network structure may underlie the retrieval difficulties commonly observed in older adults as a less efficient network could impair access to stored information (Cosgrove et al., 2021).

Different factors may modulate memory formation such as anxiety and depression, sleep quality and emotional content (Kizilbash et al., 2002; Bolton and Robinson, 2017; Cousins and Fernández, 2019; Williams et al., 2022). State anxiety is deeply related to stress (Racic et al., 2017). Stress is a physiological response to a demand or challenge and it carries feelings of physical and emotional tension (Aneshensel, 1992). Acute stress can engage a “memory formation mode,” in which rapid catecholamine and non-genomic glucocorticoid actions facilitate the encoding and early consolidation of stress-relevant information, while suppressing competing cognitive processes. This is followed by a delayed “memory storage mode,” where genomic glucocorticoid actions shield the consolidation of those memories by inhibiting new encoding or task-irrelevant information (Schwabe et al., 2012). Nonetheless, prolonged exposure to high stress can overload prefrontal cortex circuits, disrupting executive and memory functions (Arnsten, 2009; Arnsten et al., 2015). Such chronic dysregulation of prefrontal control has also been linked to the development of anxiety disorders (Patriquin and Mathew, 2017).

Depression has been linked to episodic memory impairments, particularly in the encoding and retrieval of specific details, often accompanied by a bias toward negative information (Bradley et al., 1996; Colombel, 2007; Gotlib and Joormann, 2010). These deficits have been associated with hippocampal atrophy and altered amygdala reactivity (MacQueen and Frodl, 2011; Dillon and Pizzagalli, 2018), changes that are thought to result from chronic stress and prolonged dysregulation of the hypothalamic–pituitary–adrenal (HPA) axis (Lupien et al., 2009).

Sleep is central to memory consolidation, with slow-wave sleep promoting the stabilization of declarative traces via hippocampal-neocortical interactions and supporting their reactivation, transfer, and redistribution to long-term cortical stores (Rasch and Born, 2013). According to the synaptic homeostasis hypothesis, slow-wave activity is particularly critical for downscaling synaptic strength accumulated during wakefulness, thereby preserving network efficiency and facilitating consolidation (Tononi and Cirelli, 2014).

In addition, emotional memories are better encoded as well as consolidated than neutral ones (Cahill and McGaugh, 1998; Hamann et al., 1999; Dolcos et al., 2012). This advantage has been attributed to the modulatory role of the amygdala and its interaction with hippocampal networks, which enhances the prioritization of emotionally salient information during memory formation and stabilization (Phelps, 2004). Moreover, beyond the strength of the memory trace, emotional valence also influences its organization. The temporal order of events is remembered with greater accuracy when the episodic content carries negative emotional value (D’Argembeau and Linden, 2005) and this effect is favored during sleep, a state that selectively supports the consolidation and reorganization of emotional experiences within episodic memory (Groch et al., 2011).

Regarding facial recognition, two meta-analyses have demonstrated that older adults perform worse than younger adults in lineup tasks (Fitzgerald and Price, 2015; Erickson et al., 2016). This decline is primarily attributed to a more liberal response criterion, where older adults are more likely to identify a face as familiar, even when it is not. However, a recent study by Sauerland et al. (2025) found that this effect is evident only in target-present lineups. The study proposes that older adults’ reduced associative and strategic abilities increase the likelihood of mistakenly identifying a familiar face as the perpetrator.

The COVID-19 pandemic led to a global mental health deterioration (Salari et al., 2020; Serafini et al., 2020; Etchevers et al., 2021; Nelson and Bergeman, 2021), with young adults aged 16 to 30 being the most affected (Pierce et al., 2020; Solomou and Constantinidou, 2020; Zhou et al., 2020; Belot et al., 2021). Not only were anxiety and depression levels in young adults higher during the pandemic, but they also increased significantly afterward, raising global concerns about the long-term negative effects of the COVID-19 pandemic (Khubchandani et al., 2021; Wang et al., 2022).

Young adults and children faced significant challenges during the pandemic due to a lack of social interaction in educational settings, poor sleep quality, and fear of infection. Many young people had unstable jobs with income heavily reliant on these positions. Socioeconomic status also played a crucial role: lower-income individuals were disproportionately affected by job inaccessibility and limited access to healthcare, making them more vulnerable to COVID-19’s impact (McGinty et al., 2020; Etchevers et al., 2021; Racine et al., 2021; Varma et al., 2021).

Among older adults, an increase in anxiety and depression was also observed (Etchevers et al., 2021; Yildirim et al., 2021). However, their stronger coping mechanisms may have mitigated the negative effects of isolation. Despite facing a higher risk of severe outcomes or death from contracting COVID-19, they generally had fewer concerns about issues such as job loss, quarantine, or financial instability (Vahia et al., 2020; Nelson and Bergeman, 2021; Pearman et al., 2021).

Leon et al. (2022) demonstrated that in a young population, the usual enhancement that emotional memories have over neutral ones was lost during the pandemic. In the aversive condition, higher anxiety levels were linked to poorer recall of correct details on day 1, while higher depression levels correlated with selecting more incorrect images on day 8 suggesting that heightened anxiety and depression during the pandemic may have impaired performance on aversive content tasks. Hjuler et al. (2025) examined children and adolescents’ (8 to 16 years old) autobiographical recollections of the COVID-19 lockdowns and found that memories with greater negative and factual content persisted over time and were associated with poorer psychological wellbeing. Although their focus on autobiographical memories differs from experimental approaches such as Leon et al. (2022), which contrasted neutral and aversive stimuli under controlled conditions, both converge in showing that the pervasive negativity of the pandemic shaped memory processes and was linked to worse psychological outcomes.

Based on this evidence, we hypothesize that the strong association between emotional variables and memory performance made the pandemic a specific disruptor of memory processes in young adults, as they were the most affected. As a result, we expect that during the pandemic younger and older adults will perform at comparable levels, with the possibility that older adults may even outperform younger ones in certain memory tasks. In contrast, in the post-pandemic period we anticipate that younger adults will regain their typical advantage and surpass older adults. To test this, we conducted a two-day experiment both during pandemic and in the post pandemic period in which we tested an episodic memory on young and older adults. On session 1, participants completed a set of anxiety, depression and sleep quality tests, watched an aversive video and performed an immediate free recall task (short-term testing). In the second session, they completed a recognition test using a lineup facial recognition paradigm, a free recall task assessing long-term memory, and a temporal order task. Episodic memory was therefore examined through free recall of event details, gist recall based on a predefined set of central elements identified by independent raters, and temporal order accuracy. In addition, free recall reports were analyzed using graph-theoretical measures to capture semantic organization, providing a complementary perspective on memory structure.

## 2 Materials and methods

### 2.1 Participants

161 residents of the Metropolitan Area of Buenos Aires, Argentina, aged 14 to 80 years, participated in the study. Two of them did not complete the experiment and were excluded from it. They were recruited through advertisements on social networks. All subjects signed an informed consent form prior to participation in the study. For participants under 18 years of age, consent was provided by a parent or legal guardian, in accordance with Argentine regulations that establish 18 as the legal age of majority. The study protocol was approved by the Alberto Taquini Biomedical Research Ethics Committee and the Human Ethical Committee of Buenos Aires University. None of the participants reported being sick during the experiment, having any psychiatric disorder, any history of neurological diseases, did not take any medication at the time of the experiments and did not suffer from any sleep disorders. Only older adults without cognitive impairment were included in this study, and to ensure this, a battery of cognitive tests was administered. No older adults were excluded due to cognitive deficit.

During the pandemic, the sample sizes were 42 for young adults (*M* = 16.93 SD = 1.85, 10 males and 32 females) and 41 for older adults (*M* = 71.36 SD = 4.84, 18 males and 23 females). In the post-pandemic period, the sample sizes were 38 for young adults (*M* = 17.31 SD = 1.74, 17 males and 21) and 38 for older adults (*M* = 65.38 SD = 4.03, 10 males and 18 females).

It is important to specify that in the present study we defined the younger group as participants aged 14–20 years. This decision was intentional, as this cohort was particularly exposed to the impact of pandemic restrictions. Previous research has shown that emerging adults aged 18–20 were strongly affected, especially due to disruptions in higher education and early employment opportunities (Etchevers et al., 2021). In addition, we extended the lower bound to include adolescents (14–17 years), given the profound disruption of secondary schooling and the transitional challenges faced during this period, which made them a relevant population to examine in the Argentine context.

### 2.2 Procedure

Data collection was conducted with independent samples at two distinct time points: during the pandemic (from June 2020 to July 2021) and post-pandemic periods (from June to December of 2023). In each period (pandemic, post-pandemic), participants were assigned to one of two groups: young adults (14 to 20 years) and older adults (65 to 80 years).

All the experiment was performed online via Google Meet platform from 9:00 h to 18:00 h.

**Figure 1:**
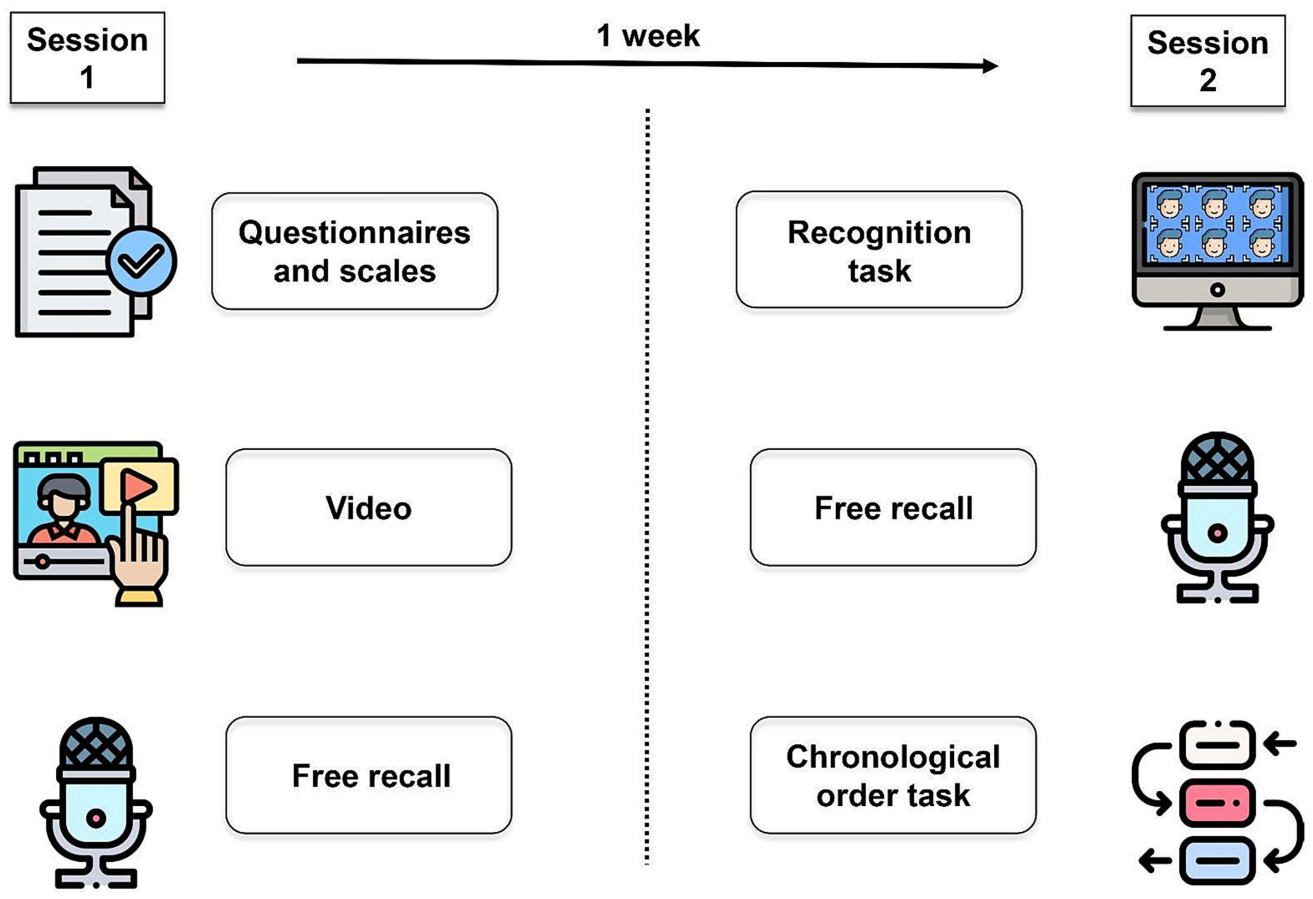
Experimental procedure. The procedure was divided into two parts: First, on session 1, the participants completed the questionnaire and mood scales. Then, they watched the aversive video and finally made a free recall of it (short-term testing). 1 week later (session 2) participants went through the last session. First, they made the recognition task and after that, they made the free recall (long-term testing). At last participants completed the chronological order task and finished the study. Icons taken from Freepik [https://www.flaticon.com/authors/freepik] from www.flaticon.com.

In session 1, participants had to complete a sociodemographic questionnaire, the symptomatological scales, and the Pittsburgh Sleep Quality Index (Buysse et al., 1989). After that, they watched a short aversive video (~ 1 min) and provided a free recall of the event. On session 2, exactly 7 days, each participant completed a lineup task where they were asked to recognize the perpetrator. After that, they gave another free recall (same as session 1) and performed a chronological order task. All the answers were recorded (Figure 1).

Because all experiments were conducted online via Google Meet, several measures were taken to standardize conditions. Participants were instructed to connect from a quiet room with stable internet, to use a laptop or desktop computer (not a phone), and to keep their screen at full size during the tasks. Audio quality was verified at the start of each session, and instructions were delivered in the same standardized format across participants. For the recognition and lineup tasks in particular, participants were specifically instructed to maintain full-screen mode to guarantee the correct visualization of the stimuli.

### 2.3 Materials

#### 2.3.1 Short aversive video

The video consisted of a young man abruptly entering a conference at the University of Buenos Aires. He started talking about university politics and was interrupted by the main speaker turning the young man furious, making him shout and throw papers that were on a desk placed at the front of the room. He finally left sobbing leaving everyone confused. The video was specifically scripted and recorded with actors during a conference setting at the University of Buenos Aires, with the purpose of being used as an experimental stimulus. This material has already been employed and validated in previous research (Urreta Benítez et al., 2021). The recording was produced at a resolution of 1,280 × 720 pixels (720p) with a frame rate of 25 fps, ensuring standard visualization quality across participants.

To determine the level of aversiveness and arousal elicited by the video, we conducted a post-hoc validation study with a separate sample of 33 participants. They rated their subjective experience using the Self-Assessment Manikin (SAM) scales for valence and arousal, ranging from 1 (extremely unpleasant / not aroused) to 5 (extremely pleasant / highly aroused). Results showed that the video was perceived as highly aversive (valence: *M* = 2.48, SD = 0.62) and moderately to highly arousing (arousal: *M* = 3.30, SD = 0.85), confirming its effectiveness as an emotional stimulus.

#### 2.3.2 Lineup

It was a 6 suspect line up where the perpetrator was always present. The perpetrator was the young man who interrupted the conference and the 5 foils were of similar age, face and complexity. All photos were black and white, numbered from left to right from one to six.

Lineup fairness: A group of participants that did not witness the event, received a brief description of the perpetrator and were asked to select the suspect from a group of 5 foils plus the perpetrator (Urreta Benítez et al., 2021). The lineup size was of 2.50 (using the acceptable lineup members technique, and considering a total of 75% minimum percentage probability expectation, Malpass and Devine, 1983; Brigham, 1990) and had a functional size of 5 (Wells et al., 1979).

Lineup instruction: “Now you are going to see a lineup with six photos, among which the person who broke into the video you saw yesterday may or may not be found. Take your time to see them. If you identify the suspect, I will ask you to tell me the number that accompanies his photo. If you consider that he is not present, tell me”.

There was no time limit for the subjects to make a decision. If participants chose the perpetrator, the answer was classified as hit (target selection); if they chose a foil or rejected the lineup, miss (foil selection plus incorrect rejection).

We used only a target-present lineup because our stimulus set included a single culprit. To properly include a target-absent condition, the optimal design would require within-subject presentation of both TP and TA lineups with the same culprit. However, this was not feasible in our case, as the video contained only one perpetrator. Our main aim was to test whether participants could successfully retrieve the culprit’s face, and the target-present lineup provided the most direct measure of this outcome.

#### 2.3.3 Free recall

Participants were asked to freely recall, in as much detail as possible, the events depicted in the video. Their responses were audio-recorded. The instruction was: *“Now I’m going to ask you to describe, in as much detail as possible, what you have watched in the video. You can include dialogues, characteristics of the people (clothes and physical characteristics), and the place. I am going to record everything you say”*.

Correct details were defined as any verifiable element of the video corresponding to objective aspects of the event, such as characters’ physical features (e.g., clothing, appearance), actions, environmental elements (objects or setting), and dialog. Each detail was counted only once, even if repeated during recall, and variations in the level of specificity (e.g., “a black short-sleeved shirt”) were treated as a single detail when referring to the same element. In addition to the total number of details, a second analysis was conducted using a list of “gist details” consisting of 10 central elements. This list was generated by four independent judges who watched the video; only the details on which they agreed were retained. Both the total number of details and the gist scores were independently coded by two trained raters, who resolved discrepancies through discussion until consensus was reached.

Finally, an exploratory network analysis was conducted on the recall reports. Three indicators were calculated: average shortest path length (ASPL), average clustering coefficient (CC), and modularity coefficient (Q). ASPL reflects the average distance across the network, CC indexes its overall connectivity, and Q captures the degree of segregation into subgroups. These measures were selected because they capture macroscopic properties of memory organization beyond node-level centrality, aligning with our aim of examining the global structure of memory representations. Prior work has shown that network efficiency and modularity are particularly informative for assessing whether information flows rapidly and coherently or tends to fragment into isolated clusters (Siew et al., 2019b; Christensen and Kenett, 2021; Ovando-Tellez et al., 2022).

#### 2.3.4 Chronological order task

The task involved five images extracted from the video, which participants had to arrange in chronological order. The images were presented simultaneously, and participants assigned a position to each. The temporal order score was calculated using two ranking methods: Relative Position Recognition (RPR) and Kendall’s Tau.

Relative Position Recognition (RPR) assesses the distance of each image from its correct chronological position. The total RPR score is the sum of these distances.

Kendall’s Tau measures the ordinal association between the participant’s order and the correct chronological order, calculating concordant and discordant pairs.

Both RPR and Kendall’s Tau were analyzed separately as dependent variables, providing complementary perspectives on temporal order performance: RPR emphasizes the degree of misplacement, while Kendall’s Tau captures sequence consistency.

#### 2.3.5 Sociodemographic questionnaire

It requested basic information about the participants such as age, gender, educational level, presence of sleep disorders and intake of any medication.

#### 2.3.6 Symptomatology scales

Beck Depression Inventory II (BDI-II). A questionnaire that contains 21 multiple choice items and measures the severity of depressive symptoms (Beck et al., 1996). State–Trait Anxiety Inventory (STAI). It consists of 40 items that evaluates two independent concepts of anxiety: Trait and State Anxiety (20 questions each). State anxiety consists of a transitory emotional state. Trait anxiety consists of a more stable and prolonged state (Spielberger et al., 1971).

#### 2.3.7 Pittsburgh sleep quality index

It is a questionnaire that assesses both quantitative and qualitative aspects of sleep quality over the month preceding its administration (Buysse et al., 1989). The PSQI relies on self-reported measures rather than objective methods like polysomnography to evaluate sleep quality.

#### 2.3.8 Cognitive deficit battery tests

Cognitive screening was conducted to ensure that all participants met the minimum criteria for inclusion. During the pandemic period, participants were evaluated with the Signoret Mnésic Battery, adapted and validated for Argentina (Leis et al., 2018). In the post-pandemic period, cognitive evaluation was performed using the Addenbrooke’s Cognitive Examination – Third Version (ACE-III), also adapted for Argentina (Bruno et al., 2020). Older adults were included only if their performance was within the normal range of each instrument, defined by the validated Argentinian cutoffs (≥11 for the Signoret and ≥86 for the ACE-III).

The use of these two instruments was based on their availability, suitability for remote administration, and validated local adaptations, which guaranteed cultural and linguistic appropriateness. In all cases, participants met the established cognitive inclusion criteria, and no individuals were excluded based on test performance.

## 3 Reports preprocessing and graph analysis

### 3.1 Text preprocessing

We performed standard text preprocessing on participant reports using the R packages tm (Feinerer et al., 2008; R Core Team, 2021), which included converting text to lowercase, removing punctuation, whitespace, numbers, stopwords as well as reducing the matrix density (sparse matrix). This approach aimed to serve the initial task of text cleaning (Kwartler, 2017).

### 3.2 Semantic network estimation

To estimate the semantic network, we employed a correlation-based method that constructs networks based on response co-occurrence within the binary response matrix. Specifically, we applied the Triangulated Maximally Filtered Graph (TMFG), a method that enhances network clarity by filtering out weaker connections and highlighting only significant associations. For the association measure, we used cosine similarity, which provides values between 0 and 1, ensuring only positive associations among nodes and thereby reducing noise from negative connections. This approach yields a clean, interpretable association matrix that is well-suited for analyzing response patterns (Massara et al., 2016; Christensen and Kenett, 2021).

## 4 Statistical analysis

Statistical analysis was carried out using the IBM software SPSS Statistics 25. Scores of the symptomatological scales (BDI, STAI and PQSI) were taken as total values (the sum of all test items).

A 2×2 between-subjects ANOVA with time (pandemic vs. post-pandemic situation) and age (young vs. older adults) as fixed factors was conducted to compare anxiety, depression, and sleep quality.

For the free recall task, the number of gist and true details were analyzed using linear mixed-effects models. The models included session (session 1 vs. session 2) as a within-subjects factor, and group (young vs. older adults) and time (pandemic vs. post-pandemic) as between-subjects factors. In addition, symptomatological scales and sleep quality measures were entered as covariates. Participant ID was modeled as a random effect to account for repeated measures.

For the order task, a series of 2 × 2 ANCOVAs were conducted, with *time* (pandemic vs. post-pandemic) and *group* (young adults vs. older adults) as between-subjects factors, and order measurements (Relative Position Recognition and Kendall’s tau) as dependent variables. Symptomatological scales and sleep quality measures were included as covariates. Simple effects analyses were conducted for all significant interactions.

Regarding the facial recognition task across different temporal phases (pandemic and post-pandemic) and age groups (young and older adults), we employed a logistic regression model. This model aimed to predict the binary outcome of facial recognition accuracy (1 for correct recognition and 0 for incorrect recognition) using the predictor variables of age, time period, depression and anxiety levels, and sleep quality. We also conducted a Chi-square test to assess the chance level across the different groups to determine if they recognized above chance level. All tests were performed with a fixed alpha of 0.05.

With respect to the exploratory graph analysis, average shortest path length (ASPL), Clustering Coefficient (CC), and Modularity (Q), were calculated as previously described and used for statistical analysis and group comparisons. To compare these measures between younger and older adults across time periods, we applied the bootstrap method (Christensen and Kenett, 2021). This approach involved re-estimating the network 1,000 times through resampling, generating sampling distributions for ASPL, CC, and Q based on empirical data alone. These distributions enabled statistical comparisons across groups by applying an analysis of covariance (ANCOVA), with the number of edges included as a covariate. Adjusting for edge count as a covariate accounts for a potential confounding factor that may otherwise impact network measure comparisons between groups.

The statistical model was defined as follows:


$$\begin{array}{l} \text{Model}:\text{Network Measure}\sim \text{Edges}+\text{Conditions}\;(\text{Group})\\ \times \text{Session}\;(1\;\text{and}\;2)\;x\;\text{Time}\;(\text{COVID}‐\text{POSTCOVID}) \end{array}$$

Additionally, we calculated Cohen’s d to determine effect size (Cohen, 2013). All network estimation and statistical procedures were implemented using the SemNeT package in R (Christensen and Kenett, 2021).

## 5 Results

### 5.1 Symptomatological scales and sleep quality

In relation to anxiety levels, there was a significant interaction between “time” and “group” [*F*_time*group_(1,154) = 4.489, *p* = 0.036]. Thus, we performed simple effects analyses of “group” within each level of “time.” During the pandemic period, there was a significant simple effect of “group” [*F* (1,154) = 5.209, *p* = 0.024], where the younger group had higher anxiety levels compared to the older group. However, in the post-pandemic period, there was no significant difference [*F* (1,154) = 0.568, *p* = 0.452]. Additionally, we performed simple effects analyses of “time” within each level of “group.” We found a significant reduction in anxiety levels from the pandemic to the post-pandemic period only in the younger group [*F* (1,154) = 20.969, *p* < 0.001], while there was no significant change in the older group [*F* (1,154) = 2.383, *p* = 0.125].

As for depression levels, there was a significant effect of “group” [*F* (1,154) = 16.821, *p* < 0.001], indicating that younger adults presented higher values in general than older adults. No significant main effect of “time” was found [*F* (1,154) = 0.035, *p* = 0.851], nor a significant “time” x “group” interaction [*F* (1, 154) = 3.480, *p* = 0.064].

Finally, there was no significant differences between groups in the sleep quality measurements [*F*_group_(1,154) = 0.323, *p* = 0.571], no significant effect of “time” [*F*_time_(1,154) = 0.402, *p* = 0.527] and no significant interaction between “time” and “group” [*F*_time*group_(1,154) = 2.207, *p* = 0.139; Table 1].

**Table 1:** Symptomatology scales and sleep quality.

	Younger adults		Older adults	
	Pandemic	Post pandemic	Pandemic	Post pandemic
Anxiety (STAI)	42.9 ± 1.2*	34.8 ± 1.3**	39.0 ± 1.2*	36.2 ± 1.3
Depression (BDI)	14.9 ± 1.2*	12.8 ± 1.3*	7.4 ± 1.2*	10.0 ± 1.3*
Sleep quality (PITTSBURGH)	5.7 ± 0.5	6.8 ± 0.5	6.8 ± 0.5	6.3 ± 0.5

Mean scores (± standard error) for anxiety, depression levels, and sleep quality index are presented for younger and older adults across pandemic and post-pandemic times. *p < 0.05, ** *p* < 0.01. Values marked with asterisks indicate significant differences between groups or across time. No significant differences were found for sleep quality.

Thus, younger adults presented higher depression and anxiety values in comparison with older adults. Regarding anxiety, younger adults were more affected during the pandemic period, however, anxiety values did not differ between groups in the post-pandemic period. Lastly, no differences were found regarding sleep quality across groups (Figure 1).

### 5.2 Memory tasks

#### 5.2.1 Free recall

**Figure 2:**
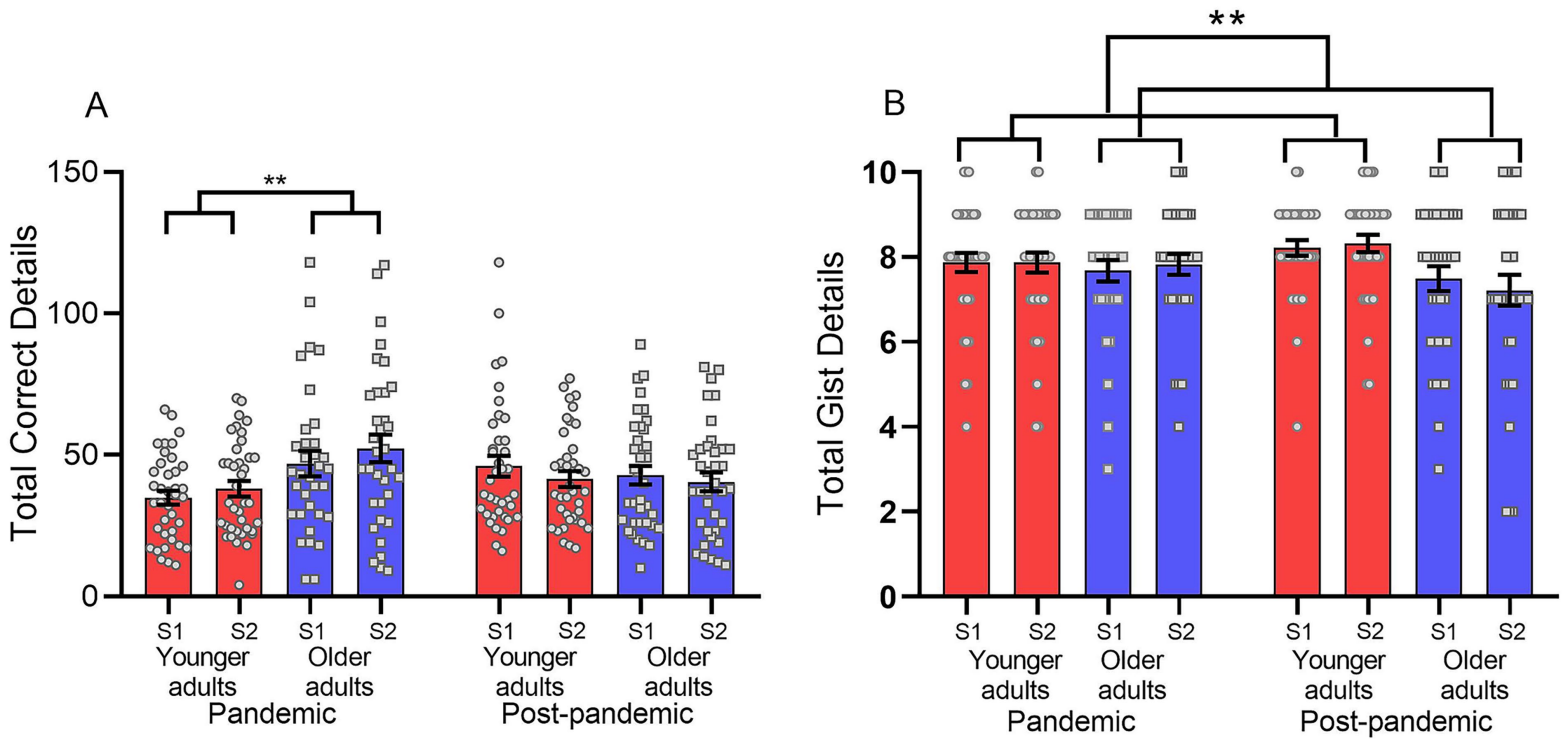
**(A)** Total correct details and **(B)** Total gist reports recalled by younger and older adults during the pandemic and post-pandemic periods. Results are shown separately for Session 1 (S1) and Session 2 (S2). The y-axis represents the total number of recalled details (Mean ± SEM). Error bars indicate standard error of the mean.

The multilevel model showed no significant interaction between “session,” “time,” and “group” [*F*_session × time×group_(1,282.9) < 0.001, *p* = 0.994], between “session” and “time” [*F*_session × time_(1,282.9) = 2.47, *p* = 0.117], or between “session” and “group” [*F*_session × group_(1,282.9) = 0.20, *p* = 0.655]. There was also no main effect of “session” [*F*_session_(1,282.9) = 0.03, *p* = 0.854]. However, there was a significant interaction between “time” and “group” [*F*_time × group_(1,282.9) = 10.12, *p* = 0.002; Figure 2A]. Thus, we performed pairwise comparisons of the estimated marginal means to study the effect of “group” within each level of “time.” We observed that during the pandemic, older adults recalled significantly more details (*M* = 51.49, SE = 2.70) than younger adults (*M* = 36.33, SE = 2.59), ΔM = 15.16, SE = 4.75, *p* = 0.006, 95% CI [−22.54, −3.77]. In the post-pandemic period, no significant group differences were observed (older: M = 41.45, SE = 2.46; younger: *M* = 42.42, SE = 2.54), ΔM = −0.97, SE = 4.65, *p* = 0.650. Regarding the pairwise comparisons of “time” within each “group,” younger adults showed no significant differences between the pandemic and post-pandemic conditions [ΔM = +6.09, SE = 4.61, *p* = 0.116; *F*_younger_(1,143) = 2.51, *p* = 0.116]. Likewise, older adults did not differ significantly across periods (ΔM = −10.05, SE = 4.78, *p* = 0.098; F_older_(1,143) = 2.78, *p* = 0.098).

This may indicate that the pandemic context had a stronger adverse effect on younger adults’ ability to encode information, which in turn resulted in fewer details being recalled at test (Figure 2A). This pattern suggests that older adults were less impacted by the pandemic context, whereas younger adults showed a greater decline in overall memory performance under those conditions.

For gist reports, the multilevel model revealed no significant interaction between “session,” “time,” and “group” [*F*_session × time×group_(1,279.1) = 0.53, *p* = 0.469], between “session” and “time” [*F*_session × time_(1,279.1) = 0.19, *p* = 0.665], or between “session” and “group” [*F*_session × group_(1,279.1) = 0.10, *p* = 0.752], and no main effect of “session” [*F*_session_(1,279.1) < 0.01, *p* = 0.980]. However, there was a significant interaction between “time” and “group” [*F*_time × group_(1,280.1) = 4.53, *p* = 0.034]. Thus, we performed pairwise comparisons of the estimated marginal means to study the effect of “group” within each level of “time.”We observed that during the pandemic there were no group differences (younger: *M* = 7.91, SE = 0.19; older: *M* = 7.82, SE = 0.20; ΔM = 0.08, SE = 0.28, *p* = 0.760), whereas in the post-pandemic period younger adults recalled significantly more gist details (*M* = 8.19, SE = 0.19) than older adults [*M* = 7.32, SE = 0.18; ΔM = 0.88, SE = 0.26, *p* = 0.001, 95% CI (0.37, 1.39); Figure 2B]. Regarding the pairwise comparisons of “time” within each “group,” younger adults did not differ significantly between the pandemic (*M* = 7.91, SE = 0.19) and the post-pandemic period (*M* = 8.19, SE = 0.19; ΔM = 0.28, SE = 0.27, *p* = 0.310). In contrast, older adults recalled significantly fewer gist details in the post-pandemic period (*M* = 7.32, SE = 0.18) than during the pandemic [*M* = 7.82, SE = 0.20; ΔM = −0.50, SE = 0.26, *p* = 0.048, 95% CI (−0.99, −0.01)].

None of the covariates (state anxiety, depressive symptoms, or sleep quality) showed significant effects (all ps > 0.11).

Overall, these results indicate that younger adults retained an advantage in gist memory, but this difference emerged specifically in the post-pandemic condition. During the pandemic, both groups performed similarly, suggesting that younger adults encoded fewer gist details under those adverse conditions, which in turn reduced the amount recalled at test.

#### 5.2.2 Order task

A series of ANCOVAs were conducted to examine the effects of “time” (pandemic vs. post-pandemic) and “group” (younger vs. older adults) on order performance (Relative Position Recognition and Kendall’s tau), while controlling for symptomatology and sleep quality as covariates.

For Relative Position Recognition (RPR), neither the main effects of “time” [*F*_time_(1,148) = 0.15, *p* = 0.701] and “group” [*F*_group_(1,148) = 0.03, *p* = 0.983], nor their interaction [*F*_time × group_(1,148) = 0.51, *p* = 0.477], were significant. None of the covariates accounted for additional variance (all ps > 0.49).

For Kendall’s tau, neither the main effects of “time” [*F*_time_(1,148) = 0.05, *p* = 0.827] and “group” [*F*_group_(1,148) = 1.18, *p* = 0.279], nor their interaction [*F*_time × group_(1,148) = 0.70, *p* = 0.405], were significant. None of the covariates reached significance (all ps > 0.43).

Overall, no significant effects of time, group, or their interaction were observed on Relative Position Recognition performance (both RPR and Kendall’s tau), and none of the covariates contributed significantly to the models.

#### 5.2.3 Facial recognition task

In relation with the logistic regression analysis only age group and sleep quality emerged as significant factors. Specifically, younger adults had a higher likelihood of recognition, *OR* = 2.26, 95% CI [1.13, 4.51], *p* = 0.021. In contrast, poorer sleep quality was associated with a decreased likelihood of recognition, *OR* = 0.89, 95% CI [0.79, 0.99], *p* = 0.048. Other variables such as anxiety, depression, and time (pandemic vs. post-pandemic) were not significant predictors in the final model. The overall model showed modest explanatory power, with Nagelkerke’s *R*^2^ reaching 0.086.

**Figure 3:**
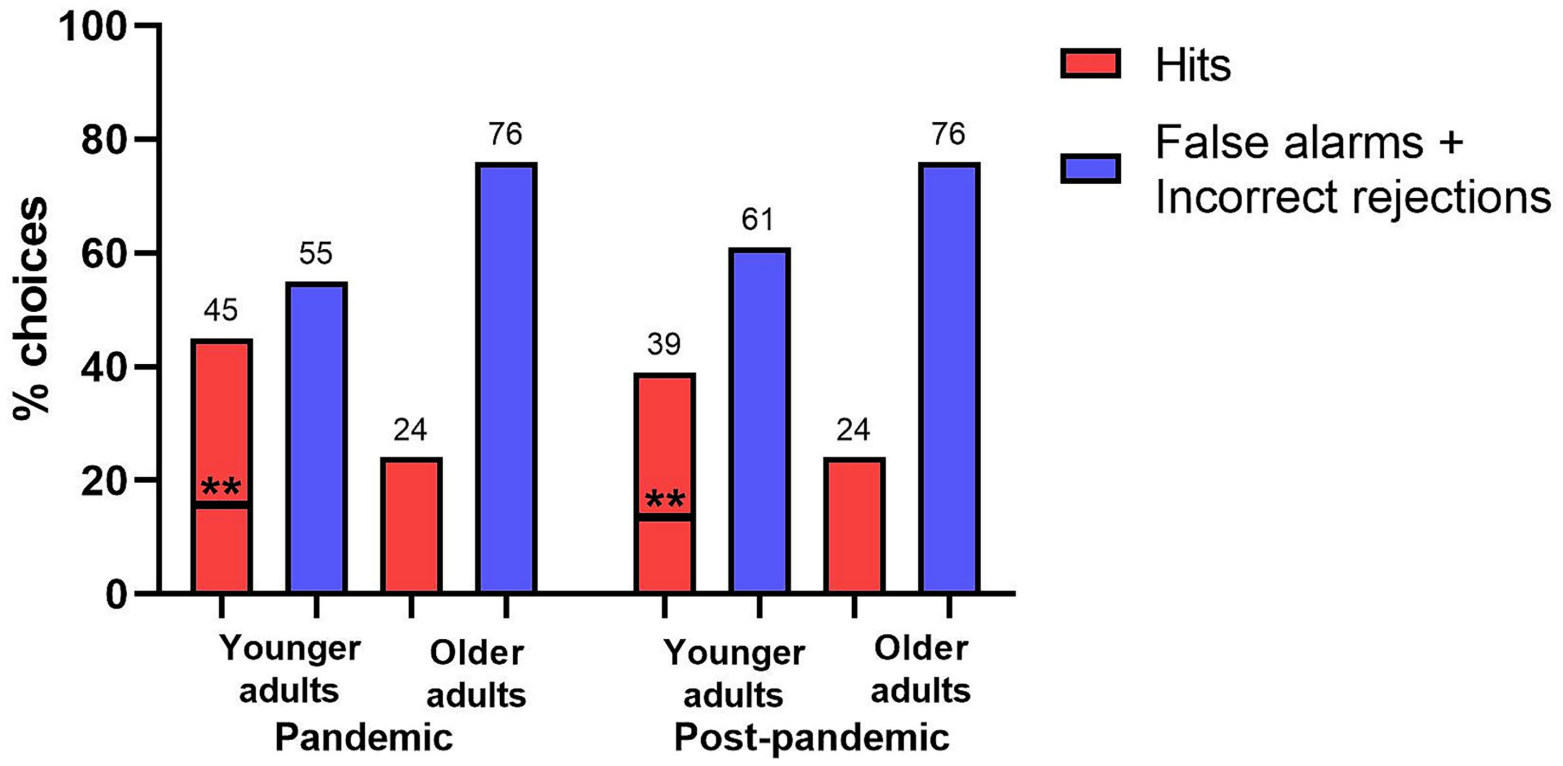
Face Recognition task with the percentage of correct choices. The dashed lines stand for the chance level (14% of correct responses). *p < 0.05.

Finally, only younger adults recognized above chance levels both during the pandemic and post-pandemic periods. If we consider that by chance 14% (one seventh) of the subjects would select the target or reject the lineup, we observed that the young adults group was significantly higher than the chance level in both times (*χ*^2^ (1) = 9.624, *p* = 0.002; *χ*^2^ (1) = 6.786, *p* = 0.009). This difference was not observed for the older adults groups (*χ*^2^ (1) = 1.242, *p* = 0.265; *χ*^2^ (1) = 1.410, *p* = 0.235; Figure 3).

#### 5.2.4 Exploratory graph analysis of the reports

**Figure 4:**
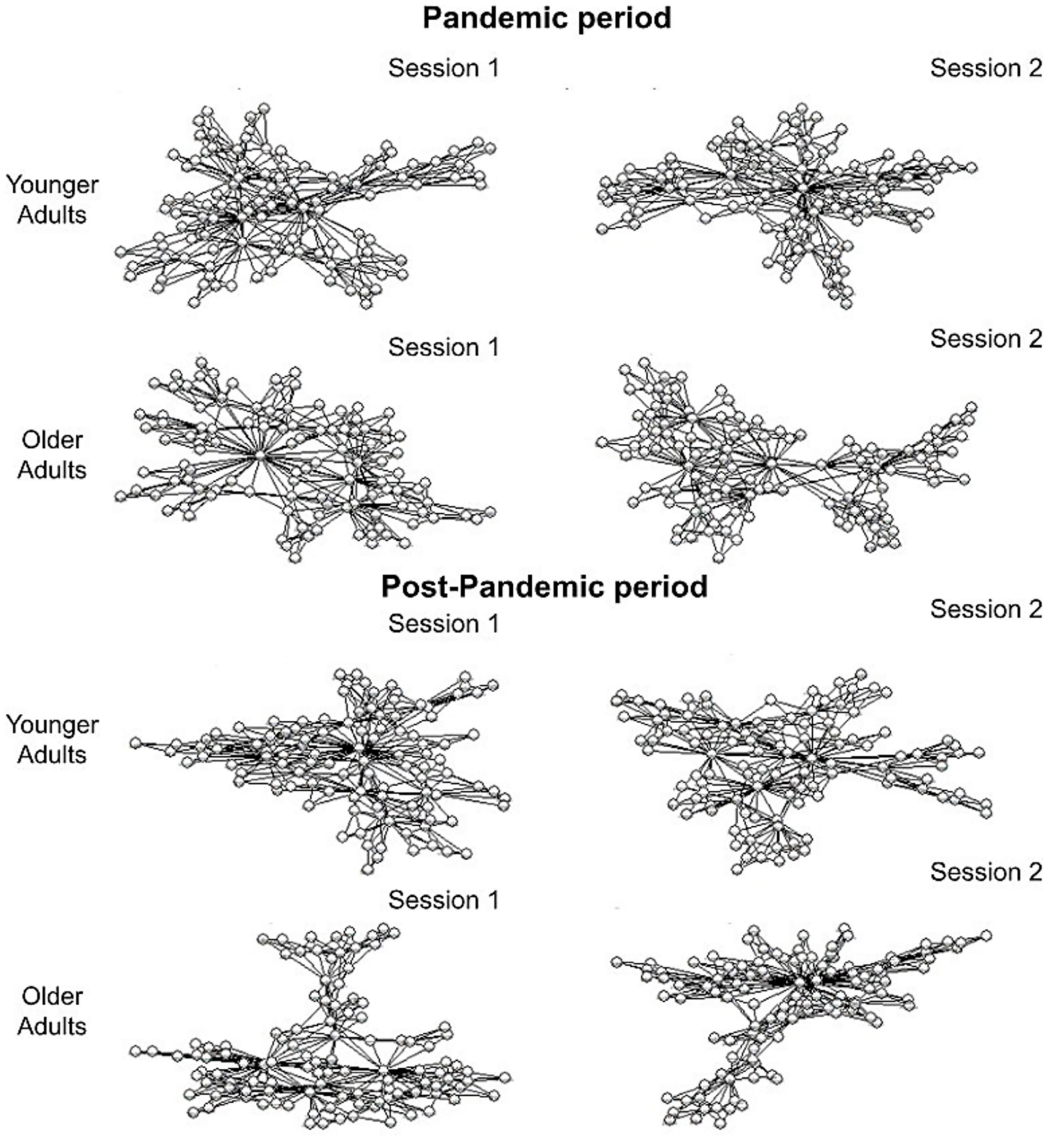
Semantic networks estimated by group, session and time period. Each dot represents a verbal response given.

Taking into account that free recall yielded two different outcomes depending on the memory variable analyzed, we further conducted an exploratory graph analysis of the reports. Unlike traditional methods, network approaches offer the advantage of uncovering emergent properties within the system, properties that remain hidden without examining the network as an integrated whole. In Figure 4 we illustrated the semantic networks resulting from the estimation in each of the groups in each time period.

Results obtained using the bootstrap method indicate that, overall, younger participants exhibit a globally more efficient network structure in terms of connectivity, distance, and modularity compared to the older adult group. Individual results for each measure are presented below.

##### 5.2.4.1 ASPL measure

Regarding the ASPL (Table 2), we found a significant interaction between “group” and “session” [*F*_session*group_ (1,143) = 32.128, *p* < 0.001], as well as a significant interaction between “group” and “time” [*F*_time*group_(1,143) = 49.476, *p* < 0.001], suggesting that the effect of the groups also depended on the time period. Lastly, we found a significant interaction between “session” and “time,” [*F*_session*time_(1,143) = 39.698, *p* < 0.001], indicating that the effect of sessions was influenced by the time period.

**Table 2:** Mean and sStandard error (SE) for ASPL.

ASPL	Pandemic		Post Pandemic	
	Session 1	Session 2	Session 1	Session 2
Younger adults	3.338 ± 0.008	3.271 ± 0.008	3.166 ± 1.200	3.207 ± 0.008
Older adults	3.541 ± 0.008	3.571 ± 0.008	3.498 ± 1.200	3.595 ± 0.008

*Post hoc* analysis of the interaction between “groups” and “time” (Table 3) showed that older adults consistently showed less efficient network organization compared to younger adults, particularly during the pandemic. Younger adults demonstrated improved network efficiency in the post-pandemic period, while older adults showed no significant changes between the pandemic and post-pandemic periods.

**Table 3:** Comparison of ASPL measures between younger and older adults in both time periods: multiple comparisons using the Tukey HSD Method. (Group*Time).

ASPL					
		Confidence interval 95%			
Group: Time period	Differences	Lower limit	Upper limit	p adjusted	d
Older A. Pandemic vs. Younger A. Pandemic	0.252	0.230	0.274	< 0.001	0.71
Younger A. Post-P vs. Younger A. Pandemic	−0.118	−0.140	−0.096	0.001	0.49
Older A. Post-P vs. Younger A. Covid	0.238	0.216	0.260	0.001	0.80
Younger Post-P vs. Older A. Pandemic	−0.370	−0.391	−0.348	0.001	1.17
Older A. Post-P vs. Older A. Pandemic	−0.014	−0.036	0.008	0.369	0.06
Older A. Post-P vs. Younger A. Post-P	0.356	0.334	0.378	0.001	1.27

##### 5.2.4.2 Clustering coefficient measure

For the clustering coefficient (CC) (Table 4), the interaction between “group,” “time” and “session” was significant [*F*_session*time*group_(1,143) = 6.725, *p* < 0.001].

**Table 4:** Mean and Standard error (SE) for CC.

CC	Pandemic		Post Pandemic	
	Session 1	Session 2	Session 1	Session 2
Younger adults	0.719 ± 0 0.001	0.721 ± 0 0.001	0.727 ± 0 0.001	0.724 ± 0 0.001
Older adults	0.708 ± 0 0.001	0.709 ± 0 0.001	0.714 ± 0 0.001	0.708 ± 0 0.001

Post-hoc analysis of the interaction between “group” and “time,” as seen in the ASPL indicator, showed that older adults consistently exhibited lower clustering coefficients compared to younger adults. This pattern was observed across both sessions during the pandemic and the post-pandemic period (Table 5).

**Table 5:** Comparison of CC measures between younger and older adults in both time periods: multiple comparisons using the Tukey HSD Method. (Groups*Time*Session).

CC					
		Confidence interval 95%			
Group: Time period	Differences	Lower limit	Upper limit	p adjusted	d
Older A. Session 1, Pandemic vs. Younger A. Session 1, Pandemic	−0.010	−0.018	−0.009	< 0.001	0.98
Older A. Session 2, Pandemic vs. Younger A. Session 2, Pandemic	−0.018	0.013	0.010	< 0.001	1.14
Older A. Session 1, Post-P vs. Younger A. Session 1, Post-P	−0.012	−0.013	0.011	< 0.001	1,69
Older Session 2, Post-P vs. Younger A. Session 2, Post-P	−0.016	−0.017	−0.014	< 0.001	1.58

In the post-pandemic sessions, the differences between younger and older adults were even more pronounced (Table 5), suggesting that younger adults maintained stronger local connectivity over time, whereas older adults showed consistently reduced clustering coefficients regardless of the session or time period (Table 6).

**Table 6:** Mean and standard error (SE) for Q.

Q	Pandemic		Post Pandemic	
	Session 1	Session 2	Session 1	Session 2
Younger adults	0.635 ± 0 0.001	0.629 ± 0 0.001	0.622 ± 0 0.001	0.625 ± 0 0.001
Older adults	0.651 ± 0 0.001	0.655 ± 0 0.001	0.645 ± 0 0.001	0.653 ± 0 0.001

##### 5.2.4.3 Modularity coefficient measure

Finally, regarding the modularity coefficient (Q), the interaction between “group” and “session” was significant, [*F*_session∗group_(1,143) = 48.432, *p* < 0.001], as was the interaction between “group” and “time,” [*F*_time∗group_(1,143) = 23.545), *p* < 0.001], and the interaction between “session” and “time,” [F_session∗time_(1,143) = 45.529, *p* < 0.001]. Post-hoc analyses for interaction between “group” and “time” showed that older adults consistently demonstrated higher modularity compared to younger adults during the pandemic and post-pandemic periods, indicating a stronger division of their networks into distinct modules. Younger adults in the post-pandemic period showed a decrease in modularity compared to the pandemic, suggesting a reduction in the separation of their network modules over time. Younger adults in the post-pandemic period exhibited lower modularity compared to older adults during the pandemic (Table 7).

**Table 7:** Comparison of Q measures between younger and older adults in both time periods: multiple comparisons using the Tukey HSD Method.

Q					
		Confidence interval 95%			
Group: time period	Differences	Lower limit	Upper limit	p adjusted	d
Older A. Pandemic vs. Younger A. Pandemic	0.021	0.0190	0.022	0.001	0.91
Younger A. Post-P vs. Younger A. Pandemic	−0.008	−0.010	−0.007	0.001	0.33
Older A. Post-P vs. Younger A. Pandemic	0.017	0.015	0.018	0.001	0.74
Younger Post-P vs. Older A. Pandemic	−0.029	−0.031	−0.027	0.001	1.25
Older A. Post-P vs. Older A. Pandemic	−0.004	−0.005	−0.002	0.001	0.17
Older A. Post-P vs. Younger A. Post-P	0.025	0.023	0.027	0.001	1.08

(Groups*Time*Session).

Overall, the older adult group displayed a semantic network where their components were, on average, more distant from each other (ASPL in older adults > ASPL in younger adults, in both time periods and in both sessions) and less connected (CC in older adults < CC in younger adults, in both time periods and in both sessions). For the older adult group, the estimated networks also indicated greater modularity in their structure (Q in older adults > Q in younger adults, in both time periods and in both sessions). Additionally, the younger adult group showed an improvement in network efficiency from session 1 to session 2, which was not observed in the older adult group. In older adults, the global network values remained stable between session 1 and session 2. Furthermore, the differences between younger and older adults across all indicators (ASPL, CC, and Q) were smaller during the pandemic compared to the post-pandemic period, suggesting the possibility that younger adults were more adversely affected during the pandemic, with their network efficiency being closer to that of older adults during this time.

## 6 Discussion

Based on the studies mentioned above, emotional wellbeing was particularly compromised during the COVID-19 pandemic, with younger adults showing higher levels of anxiety and depression as well as disruptions in their sleep routines. This context provided a unique opportunity to investigate how episodic memory processes operate under stress. In our study, we compared younger (14–20 years) and older adults (65–80 years) across pandemic and post-pandemic periods, focusing on free recall, gist recall, facial recognition, and temporal order.

Younger adults reported higher anxiety and depression, with anxiety decreasing only after the pandemic. In free recall, older adults remembered more details during the pandemic, but this advantage disappeared post-pandemic. In gist recall, younger adults outperformed older adults only in the post-pandemic condition. In facial recognition, younger adults consistently showed higher accuracy across both periods. Finally, semantic networks in older adults were more modular and less connected, whereas younger adults exhibited greater efficiency after the pandemic.

Specifically, in the total detail free recall task, the older group performed better than the younger group during the pandemic, while this difference disappeared in the post-pandemic period. This suggests that younger adults were more affected by the pandemic situation. The cognitive challenges observed in younger adults during the pandemic might be linked to their significantly higher levels of depression and anxiety compared to older adults (Table 1). In line with these results, prior to the pandemic, depression levels were higher for older adults, while anxiety levels were higher in young adults (Figueroa, 1991; Bonicatto et al., 1998). However, the pandemic clearly altered these patterns, leaving younger adults more vulnerable in both mood variables during the pandemic period. Although we expected anxiety, depression, and sleep quality to explain differences in memory performance, our analyses did not support this. This suggests that these variables alone cannot account for the disproportionate impact of the pandemic on young adults’ memory. Instead, unmeasured contextual stressors may have played a critical role. Economic concerns and financial strain were among the strongest predictors of mental health problems during the pandemic (Chan et al., 2024), and structural models show that job loss, housing insecurity, and financial hardship mediated psychological distress (Sundaram-Stukel and Davidson, 2021). Socioeconomic status has also been linked to greater vulnerability in cognitive outcomes following COVID-19 (Aderinto et al., 2025). Factors such as household income, family members’ occupational exposure to the virus, household density, and caregiving responsibilities may therefore have interacted with emotional symptoms in complex ways, masking direct links and better explaining the observed changes in memory performance.

Regarding the gist free recall task, younger adults recalled significantly more information than older adults but this was only in the post-pandemic period, and the differences observed in the previous free recall test disappeared. These results underscore the importance of considering the fuzzy-trace theory (Reyna and Brainerd, 1998), which posits that gist details are more likely to be remembered than literal details and are less susceptible to decay over time. Gist details are quickly encoded and stored, facilitating their future retrieval. Age is of most relevance since it has been demonstrated how older adults show a general cognitive decline that leads to a lesser possibility of retrieving verbatim and gist details and they rely more on reconstructive information that could lead to the apparition of, for example, false memories (Brainerd and Reyna, 2015). Thus, the fact that this difference was only observed in the post-pandemic period suggests that the typical age-related advantage of younger adults was diminished during the pandemic but reemerged when conditions normalized, particularly when the task became more structured and focused solely on gist reports. If we take into account the gist reports measure, it reflects more controlled and structured ways of assessing episodic memory, aligning with typical age-related differences (Brainerd and Reyna, 2015). Moreover, older adults only showed an improved performance on the free recall task but no enhancement when recalling from a pre-arranged set list of details. A possible explanation could be the associative deficit hypothesis (Naveh-Benjamin et al., 2003), which suggests that older adults can retrieve general details of an event as effectively as younger adults but often fail to encode or retrieve the associations among these details. Campbell and Davis (2024) recently demonstrated how this theory aligns with findings that poor attentional control in older adults leads to increased interference during retrieval, resulting in what they term excessive associations or “hyper-binding.” This may have allowed older adults to perform better in free recall, where they could include many less relevant details, while the gist task highlighted their difficulty in recalling the target items.

Furthermore, we conducted an exploratory graph analysis because network-based measures can uncover emergent properties of the system that are not captured by criterion-based content analysis, which focuses on the presence or absence of predefined indicators in recall reports. Thus, taking in consideration all three indicators (ASPL, CC and Q), during the pandemic, the difference between the two groups was smaller, likely due to a decline in cognitive performance among younger adults. However, in the post-pandemic period, this difference increased significantly, with younger adults showing better performance than during the pandemic. These findings suggest that the pandemic had a more pronounced negative effect on the cognitive performance of younger adults.

In comparison with younger adults, older adults show higher ASPL (distance), lower CC (connectivity), and higher Q (communities-modularity). These results are consistent with previous findings, which generally show that semantic networks in older adults are more modular, that is, composed of more dispersed and less connected clusters, and more segregated, with greater separation between pairs of nodes, compared to younger adults (Dubossarsky et al., 2017; Siew et al., 2019a; Cosgrove et al., 2021; Wulff et al., 2022). This pattern reflects the natural age-related decline. In cognitive terms, higher modularity implies that concepts are stored in smaller, more isolated pockets of the network, which limits the spread of activation across concepts. Such a less efficient and less integrated network structure may contribute to the retrieval difficulties typically observed in older adults (Cosgrove et al., 2021).

In the facial recognition task, our analysis highlights the importance of age and sleep quality as key factors influencing performance. Younger adults demonstrated a higher likelihood of correct recognition, emphasizing the role of age in this cognitive domain. Poor sleep quality was associated with reduced recognition accuracy reinforcing its impact on cognitive performance. Other factors like anxiety, depression, and the period in which the study was run did not significantly affect recognition performance. Additionally, only younger adults consistently recognized above chance levels both during and after the pandemic, while older adults did not, indicating that younger adults maintained better recognition abilities across different contexts. The increased cognitive demands during the pandemic may have further strained older adults’ resources, while younger adults, despite heightened anxiety and depression, likely maintained better performance possibly due to more efficient cognitive strategies.

No significant differences were found between groups in both times regarding the episodic order task. These results do not align with the existing literature, which suggests that aging reduces memory retrieval, including the ability to organize past events in the right order (Wahlheim and Huff, 2015; Talamonti et al., 2021). The advantage younger people usually have in tasks involving the sequence of events does not seem to affect performance in this particular task. A possible explanation for the lack of difference could be that the images used captured the gist of the video, potentially leading to a ceiling effect, where both groups found the task relatively easy and older adults did not exhibit the expected decline. In line with this interpretation, future studies should consider increasing task difficulty by incorporating a greater number of images, including less central or more ambiguous details, or extending the sequence length. Such refinements would reduce ceiling effects and provide a more sensitive assessment of age-related differences and contextual influences on temporal order memory.

Thus, this study contributes to a better understanding of how major societal stressors, such as the COVID-19 pandemic, differentially affect episodic memory processes in young and older adults. From a theoretical perspective, they provide insights into the mechanisms through which stress and negative emotional contexts modulate encoding and retrieval, adding to existing models of memory and stress. From an applied perspective, these results highlight the need to consider age-related differences when evaluating eyewitness memory and psychological wellbeing in crisis contexts. Notably, our data suggest that young adults may be particularly vulnerable during periods of prolonged stress, underscoring the importance of prioritizing their mental health and implementing timely interventions to mitigate long-term cognitive and emotional consequences. These consequences must be carefully evaluated to mitigate potential harm to critical processes such as eyewitness testimony, where errors could lead to severe outcomes, such as the wrongful conviction of an innocent person.

This study has several limitations that should be acknowledged. First, the initial phase of data collection took place during the COVID-19 pandemic, which imposed important constraints on the design and implementation of the study. One limitation of the present study is that different cognitive screening tools were administered across data collection periods (the Signoret Mnésic Battery during the pandemic and the ACE-III in the post-pandemic phase). This methodological inconsistency does not allow for direct comparison of raw cognitive scores between groups. However, in both cases the screenings served the same purpose of ensuring that all participants met minimum cognitive inclusion criteria, and no participants were excluded based on their performance. Thus, while this reduces cross-group comparability at the screening level, it does not affect the validity of the main findings regarding memory performance. Second, because all testing was conducted online, there may have been uncontrolled variance in participants’ environments and viewing conditions. Third, the absence of a target-absent lineup constrains the generalization of our findings to facial recognition scenarios that more closely resemble real forensic settings. In addition, the sample was drawn exclusively from the Metropolitan Buenos Aires area, which may introduce sampling bias and reduce the generalizability of the results. Another limitation of this study is that socioeconomic status and educational level were not systematically recorded. Although age and gender were collected, these variables were not incorporated into the analyses. Future studies should examine their potential influence on memory outcomes.

## Data Availability

The raw data supporting the conclusions of this article will be made available by the authors, without undue reservation.
